# Five-year overall and specific survival of breast cancer in great Cuiaba (MT), Brazil

**DOI:** 10.1590/1980-549720250010

**Published:** 2025-03-03

**Authors:** Jânia Cristiane de Souza Oliveira, Noemi Dreyer Galvão, Amanda Cristina de Souza Andrade, Ageo Mário Cândido da Silva

**Affiliations:** I Universidade Federal de Rondonópolis, School of Health Sciences – Rondonópolis, (MT), Brazil.; II Universidade Federal de Mato Grosso, Institute of Public Health – Cuiabá (MT), Brazil.; III Mato Grosso State Health Department – Cuiabá (MT), Brazil.

**Keywords:** Women's health, Breast neoplasms, Survival analysis, Health information systems

## Abstract

**Objective:**

To analyze the overall and cancer-specific five-year survival rates for female breast cancer in Greater Cuiabá, Mato Grosso, Brazil.

**Methods:**

A non-concurrent, population-based cohort study using the Population-Based Cancer Registry of Greater Cuiabá (Cuiabá and Varzea Grande), including women diagnosed with breast cancer from 2008 to 2013, followed through 2018 in the regional mortality database. The sample consisted of a total of 1,220 women. Five-year survival analysis was performed using Kaplan-Meier curves and the Cox proportional hazards regression model, computing hazard ratios for variable estimation. Survival curves were compared using the log-rank test (p<0.05). Probabilistic linkage technique by the RecLink III software and survival analysis were conducted using STATA software version 12.0.

**Results:**

There was no statistical difference between the overall (OS) and cancer-specific survival (SS) rates (OS 78.0%, 95%CI 75.6–80.2; SS 81.0%, 95%CI 78.7–83.2). Women with lower educational levels (OS=58.33%; SS=64.89%) and those without a partner (OS 64.81%; SS 70.41%) exhibited poorer survival.

**Conclusion:**

This study demonstrates that educational level and marital status significantly impact both overall and cancer-specific survival rates for female breast cancer. There is a need to propose policies that address the profile of women with lower survival rates.

## INTRODUCTION

Breast cancer is the most prevalent cancer among women globally. In 2022, the estimated incidence rate was 46.8/100,000 women. By 2045, the number of new cases is projected to increase by 46.5%, with over 3.3 million women expected to be diagnosed with the disease. In South America, the projected increase in new cases for the same period is 47.8%^1^.

Globally, breast cancer is also the leading cause of cancer-related deaths in women, with a mortality rate of 12.7/100,000 women reported in 2022. By 2045, cancer-related deaths are projected to increase by 59.1% worldwide. In South American countries, the estimated mortality rate in 2022 was 13.8/100,000 women, with a projected 63.3% increase in deaths by 2045^1,2^.

In Brazil, breast cancer is the most prevalent cancer among women across all regions of the country. For the three-year period from 2023 to 2025, the age-adjusted incidence rate was estimated at 66.5/100,000 women, with a projected increase of 47.6% by 2045. In 2022, the age-adjusted mortality rate was estimated at 13.9/100,000 women, with breast cancer mortality in the country expected to rise by 61.9% by 2045^2,3^.

Cancer survival rates in the Americas exhibit significant heterogeneity. In North America and Costa Rica, survival rates are among the highest (≥85.0%). Within Latin America, disparities are evident. For example, Argentina, Peru, and Puerto Rico report survival rates ranging from 80.0 to 84.0%, while countries such as Brazil, Colombia, Cuba, and Ecuador have survival rates between 70.0 and 79.0%^4^.

In addition to early diagnosis, several factors contribute to improved survival rates in women with breast cancer. These include positive hormone receptor status, a diagnosis of postmenopausal breast cancer, the absence of the triple-negative subtype, white race/skin color, having a partner, higher socioeconomic status, and access to health insurance^5-7^.

Another contributing factor is that, despite being a priority in the state's Oncology Plan, the control of female breast cancer remains a challenge. This is primarily due to issues in improving network coordination and the need for increased investment to reduce delays between initial suspicion, diagnosis, and the initiation of treatment. Additionally, the study aimed to provide insights into the current situation to inform future actions for more effective disease control^8^.

The aim of the study was to analyze five-year overall survival (OS) and specific survival (SS) rates for female breast cancer in a cohort from 2008 to 2013, based on the population of Greater Cuiabá, Mato Grosso, Brazil.

## METHODS

A non-concurrent, population-based cohort was established, comprising women diagnosed with breast cancer and recorded in the Population-Based Cancer Registry (*Registro de Câncer de Base Populacional –* RCBP/Cuiabá). This registry includes data from the cities of Cuiabá and Várzea Grande in Mato Grosso State, Brazil, covering the period from January 2008 to December 2013. Only incident cases (definitive indicator "true") were included, while cases of *in situ* breast cancer were excluded. Follow-up was conducted passively through the Mato Grosso Mortality Information System (*Sistema de Informação sobre Mortalidade* – SIM) for a period of five years.

The RCBP Cuiabá coverage includes the municipalities of Cuiabá and Várzea Grande, the two largest municipalities in the state, contiguous, with a population of almost one million inhabitants according to the 2022 census, with 650,877 inhabitants in Cuiabá^9^ and 300,078 in Várzea Grande; the urban conglomerate is called Greater Cuiabá^10^.

The RCBP Cuiabá was established in 1999 by the State Department of Health of Mato Grosso. However, the data became outdated after 2007. In response, a partnership was initiated in 2016 with Universidade Federal de Mato Grosso to update the databases, a process that continued until March 2021. Currently, the RCBP Cuiabá relies on 38 health establishments as reporting sources, including hospitals and diagnostic support clinics^11^. Among these, three hospitals are registered with INCA as High Complexity Oncology Units (*Unidade de Alta Complexidade em Oncologia* – Unacon)^12^. However, the Hospital Cancer Registries that contribute to the RCBP Cuiabá still exhibit significant incompleteness in key variables, such as education, marital status, tumor-node-metastasis (TNM) classification, staging, and disease status at the conclusion of the first treatment^13^.

**Figure 1:**
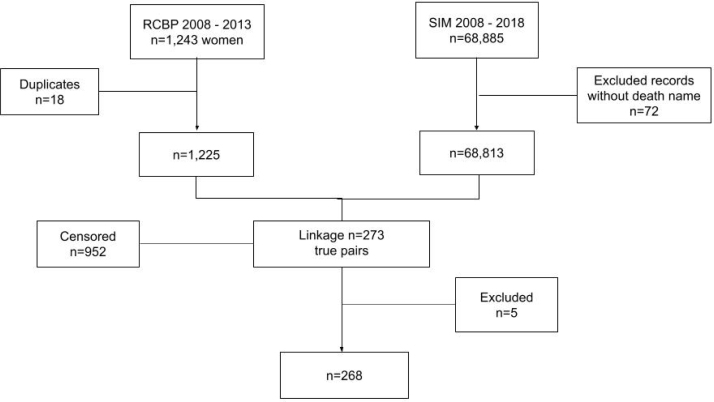
Flowchart.

Regarding the preparation of the databases, the SIM database was cleaned prior to pairing, with cases where the deceased's name was listed as "blank," "unknown," "ignored," or "indigent" being excluded, resulting in 72 exclusions. In the RCBP database, duplicates were removed. Records with identical data for patient name, mother's name, gender, date of birth, disease code, and date of diagnosis were classified as duplicates, leading to the exclusion of 18 records (Figure 1).

The probabilistic linkage between the RCBP and SIM databases was performed according to the three steps proposed by Coeli and Camargo Jr.^14^: standardization, which involved standardizing common fields for pairing; blocking, using the gender variable; and, finally, pairing by constructing concordance scores based on the variables of patient name (death), mother's name, and date of birth.

Based on the probabilistic linkage between the databases, 273 pairs were identified, all of which were confirmed as true. Of these, five records lacked information on "age" and "date of birth," and one record had an age of zero (with the date of birth matching the date of diagnosis). After attempting to recover this data from the Unified Health System User Registration System (*Sistema de Cadastramento de Usuários do Sistema Único de Saúde* – CADSUS), it was only possible to retrieve the record with an age of zero. The other records without age information were excluded from the database, resulting in a total of 1,220 records: 268 failures (women who experienced the outcome of death during the study period) and 952 censored cases (women who did not experience the outcome of death during the study period)^15^ (Figure 1).

The dependent variable was the time from the women's entry into the study (date of breast cancer diagnosis) to death from any cause (OS) or from breast cancer specifically (SS). Such studies are referred to as survival analysis^15^. Survival time was measured in months, and women who did not experience the event of interest during the study period were considered censored.

The following independent variables were analyzed: age group (20–49 years, 50–69 years, ≥70 years); race/skin color (yellow, white, brown, black, and no information); marital status (with partner, without partner, and no information); education level (0–7 years of study, 8+ years of study, and no information); municipality of residence at the time of diagnosis (Cuiabá, Várzea Grande); diagnostic method (histology of the primary tumor, others); and morphology (infiltrating ductal carcinoma, others).

An initial assessment was performed using absolute and relative frequencies. The variables education, marital status and race/skin color presented 42.70, 26.48, and 9.75% of missing data, respectively. Given the impossibility of multiple imputation of missing data due to the reduced number of independent variables available and with complete data^16^, the multiple model was performed only with complete data (excluding those without information) (n=645).

Survival probabilities and their respective 95% confidence intervals (95%CI) were calculated for each independent variable. The Kaplan-Meier estimator was employed to estimate survival curves for the event of interest, and the curves were compared using the log-rank test, with a significance level of 5%.

The effect of independent variables on survival was estimated using the Cox proportional hazards regression model, with hazard ratios (HR) and their corresponding 95%CI computed. The variables morphology and diagnostic method did not satisfy the Cox proportional hazards assumption; therefore, the multiple model was stratified by these variables. The proportional hazards assumption was assessed using Schoenfeld residuals, ensuring no rejection of the null hypothesis (p > 0.05)^16^.

The probabilistic linkage was performed using Link Plus 2.0 software, while survival analysis was conducted using Stata 12.0.

The study adheres to the principles outlined in Resolution 466/2012 of the National Health Council. It is part of the matrix project entitled "Cancer and its associated factors: analysis of population and hospital records in Cuiabá-MT," which was approved by the Ethics Committee of the Júlio Muller University Hospital and the Ethics Committee of the School of Public Health of the State of Mato Grosso — State Secretariat of Health of Mato Grosso.

## RESULTS

Among the 1,220 women included in the study, 80.08% resided in Cuiabá, 59.67% identified as non-white, 29.75% had more than eight years of schooling, and 36.80% were unpartnered. A total of 92.62% were diagnosed through histology of the primary tumor, and 78.85% had invasive ductal carcinoma morphology. The majority of women were aged 50 years old or older (60.33%), with a mean age of 54 years and a median age of 53 years (interquartile range: 45 to 63 years), with ages ranging from 21 to 99 years (Table 1).

**Table 1:** Characterization of women diagnosed with breast cancer. Cuiabá (MT), 2008–2013.

Characteristic		Total n=1,220 (%)	Death from any cause n=268 (%)	Death from breast cancer n=228 (%)	Censored n=952 (%)
Age range (years)					
	20–49	484 (39.67)	99 (36.94)	90 (39.47)	385 (40.45)
	50–69	572 (46.89)	114 (42.54)	99 (43.42)	458 (48.11)
	70 +	164 (13.44)	55 (20.52)	39(17.11)	109 (11.44)
Race/color					
	Yellow	36 (2.95)	4 (1.49)	3 (1.32)	32 (3.36)
	White	373 (30.57)	94 (35.07)	75 (32.89)	279 (33.41)
	Brown	635 (52.05)	150 (55.97)	132 (57.89)	485 (50.95)
	Black	57 (4.67)	18 (6.72)	16 (7.02)	39 (4.10)
	No information	119 (9.75)	2 (0.75)	2 (0.87)	117 (12.28)
Education level (years)					
	0–7	336 (27.54)	140 (52.24)	114 (50.00)	196 (20.59)
	8+	363 (29.75)	116 (43.28)	103 (45.18)	247 (25.95)
	No information	521 (42.70)	12 (4.48)	11 (4.82)	509 (53.47)
Marital status					
	With partner	448 (36.72)	106 (39.55)	95 (41.67)	342 (35.92)
	Without partner	449 (36.80)	158 (58.96)	129 (56.58)	291 (30.57)
	No information	323 (26.48)	4 (1.49)	4 (1.75)	319 (33.51)
Municipality					
	Cuiabá	977 (80.08)	216 (80.60)	181 (79.39)	761 (79.94)
	Várzea Grande	243 (19.92)	52 (19.40)	47 (20.61)	191 (20.06)
Diagnosis method					
	Histology of primary tumor	1,130 (92.62)	219 (81.72)	179 (78.51)	911 (95.69)
	Others	88 (7.21)	49 (18.28)	49 (21.49)	39 (4.10)
	No information	2 (0.16)	0	0	2 (0.21)
Morphology					
	Invasive ductal carcinoma	962 (78.85)	184 (68.66)	155 (67.98)	778 (81.72)
	Outros	258 (21.15)	84 (31.34)	73 (32.02)	174 (18.28)

Source: Research data; 2021.

By the end of the study, among the 1,220 women being followed, 268 had died. Of these, 228 (85.76%) deaths were due to breast cancer, 14 (5.23%) resulted from other types of cancer, and 26 (9.71%) were attributed to other identified causes.

**Figure 2:**
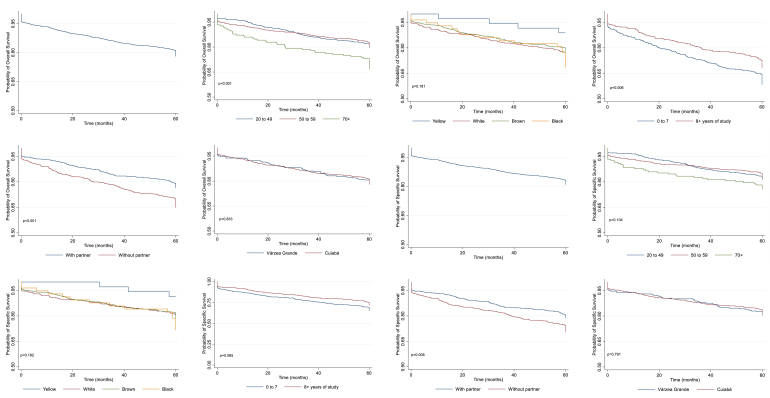
Overall Survival and Five-Year Specific Survival of Female Breast Cancer, Population-Based Cancer Registry (RCBP) — Cuiabá (MT), Brazil.

RCBP: Population-Based Cancer Registry (*Registro de Câncer de Base Populacional*); SIM: Mortality Information System (*Sistema de Informações sobre Mortalidade*).

The OS and SS rates did not show a statistically significant difference (OS: 78.03%, 95%CI: 75.60–80.25%; SS: 81.05%, 95%CI: 78.72–83.16%). In the OS analysis, women aged 70 years old or older and those with an education level of zero to seven years demonstrated lower survival rates. Additionally, women without a partner exhibited lower OS and SS rates (log-rank test, p<0.05) (Table 2 and Figure 2). Histology of the primary tumor showed the highest OS and SS rates, as did invasive ductal carcinoma regarding morphology (p<0.05) (Table 2).

**Table 2:** Overall survival functions and five-year specific survival of women diagnosed with breast cancer, according to the study variables. Cuiabá (MT), 2008–2013.

Characteristic		Overall survival % (95%CI)	Specific survival % (95%CI)
Age range (Years)			
	20–49	79.55 (75.67–82.87)	81.17 (77.37–84.40)
	50–69	80.07 (76.56–83.12)	82.51 (79.12–85.40)
	70 +	66.46 (58.68–73.12)	75.58 (68.12–81.53)
Race/color			
	Yellow	88.89 (73.05–95.68)	91.43 (75.73–97.15)
	White	74.80 (70.07–78.90)	79.47 (74.95–83.27)
	Brown	76.38 (72.88–79.49)	78.96 (75.56–81.95)
	Black	68.42 (54.66–78.79)	71.55 (57.82–81.50)
	No information	98.32 (93.45–99.58)	98.32 (93.45–99.58)
Education level (years)			
	0–7	58.33 (52.87–63.39)	62.87 (54.92–69.82)
	8+	68.04 (62.98–72.57)	73.83 (66.29–79.94)
	No information	97.66 (96.01–98.64)	97.84 (96.23–98.77)
Marital status			
	With partner	85.73 (83.06–88.01)	87.09 (84.51–89.27)
	Without partner	64.81 (60.20–69.03)	70.41 (65.87–74.47)
	No information	98.76 (96.73–99.53)	98.76 (96.73–99.53)
Municipality			
	Cuiabá	77.89 (75.16–80.37)	81.21 (78.60–83.54)
	Várzea Grande	78.60 (72.89–83.25)	80.41 (74.80–84.90)
Diagnosis method			
	Histology of primary tumor	80.62 (78.19–82.81)	83.87 (81.57–85.91)
	Others	44.32 (33.79–54.32)	44.32 (33.79–54.32)
Morphology			
	Invasive ductal carcinoma	80.97 (78.24–82.22)	83.62 (81.10–85.89)
	Others	67.44 (61.35–72.79)	71.52 (65.56–76.63)

Source: Research data, 2021.

In the adjusted Cox model, the age group variable demonstrated statistical significance only for OS. For the adjusted models, including the stratified Cox model, marital status was statistically significant for both survival rates (OS and SS). The remaining variables did not exhibit statistically significant differences (Table 3).

**Table 3:** Unadjusted and adjusted hazard ratios (HR) for overall survival and five-year specific survival of women diagnosed with breast cancer, according to variables selected for the multiple model. Cuiabá (MT), 2008–2013.

Characteristics			Non-adjusted	Adjusted analysis	
				COX Model*,‡	Stratified COX model†,‡
			HR (95%CI)	HR (95%CI)	HR (95%CI)
Overall survival					
	Age range (years)				
		20–49	1	1	1
		50–69	0.98 (0.75–1.28)	0.97 (0.73–1.28)	0.94 (0.71–1.25)
		70 +	1.78 (1.28–2.47)	1.55 (1.08–2.25)	1.29 (0.89–1.87)
	Race/color§				
		Yellow	1	1	1
		White	0.95 (0.35–2.58)	0.91 (0.33–2.49)	0.76 (0.28–2.09)
		Brown	0.81 (0.30–2.20)	0.79 (0.29–2.16)	0.67 (0.24–1.82)
		Black	1.20 (0.41–3.55)	1.09 (0.37–3.25)	0.98 (0.33–2.91)
	Education level// (years)				
		0–7	1.40 (1.09–1.79)	1.24 (0.95–1.60)	1.23 (0.94–1.60)
		8+	1	1	1
	Marital status¶				
		With partner	1	1	1
		Without partner	1.59 (1.24–2.03)	1.44 (1.11–1.87)	1.49 (1.15–1.94)
	Municipality				
		Cuiabá	1.03 (0.76–1.40)	1.02 (0.75–1.41)	1.01 (0.73–1.38)
		Várzea Grande	1	1	1
Specific survival					
	Age range (years)				
		20–49	1	1	1
		50–69	0.93 (0.70–1.24)	0.93 (0.69–1.25)	0.89 (0.66–1.21)
		+70	1.38 (0.95–2.01)	1.22 (0.80 –1.87)	0.99 (0.64–1.51)
	Race/color§				
		White	1	1	1
		Yellow	0.99 (0.31–3.17)	0.97 (0.30–3.11)	0.80 (0.25–2.58)
		Brown	0.95 (0.30–2.98)	0.92 (0.29–2.91)	0.76 (0.24–2.41)
		Black	1.42 (0.41–4.87)	1.34 (0.39–4.62)	1.23 (0.35–4.24)
	Education level// (years)				
		0–7	1.32 (0.88–1.97)	1.17 (0.88–1.55)	1.16 (0.87–1.53)
		8+	1	1	1
	Marital status¶				
		With partner	1	1	1
		Without partner	1.45 (1.11–1.89)	1.37 (1.03–1.81)	1.44 (1.09–1.91)
	Municipality				
		Cuiabá	0.96 (0.69–1.32)	0.97 (0.69–1.36)	0.95 (0.68–1.35)
		Várzea Grande	1	1	1

Source: Research data, 2021.

* Global test value for the proportional hazards assumption: p=0.733 (OS); p=0.285 (SS).

† Global test value for the proportional hazards assumption: p=0.853 (OS); p=0.754 (SS); Stratified by morphology and diagnostic method variables;

‡ n=645;

§ n=1.010;

// n=699;

¶ n=897.

## DISCUSSION

This study analyzed the OS and SS of women with breast cancer in Greater Cuiabá, Mato Grosso, Brazil. No statistical difference was observed between the two survival rates. In the multiple analysis, marital status demonstrated statistical significance for both survival rates (p<0.05).

The findings of this study align with the survival rates reported for Brazil (data from the RCBP of Aracaju, Cuiabá, Curitiba, Goiânia, Jaú, and São Paulo), analyzed by CONCORD-3^4^. This study compared the net survival of 18 cancers across 71 countries. In countries such as Denmark, France, Norway, Finland, Israel, Japan, Australia, New Zealand, Canada, the United States, and Costa Rica, survival rates for female breast cancer were higher (≥85.0%). Argentina, Peru, and Puerto Rico reported survival rates of 80.0–84.0%. Brazil was among the countries with survival rates of 70.0–79.0%, similar to Cuba, Ecuador, Bulgaria, and Poland. Another study conducted in Poland from 2000 to 2019 found a five-year survival rate of 77.3%^17^, a result comparable to the findings of this study.

A population-based study conducted in Goiânia^18^, which included women diagnosed with breast cancer between 1995 and 2003, reported a 5-year OS rate of 72.1%, lower than the rate observed in the present study. This difference may be attributed to the study period or potential changes in screening and treatment protocols for the disease over time.

Higher survival rates were observed in Spain^19^, where a cohort study conducted from 2000 to 2007 analyzed all primary tumors diagnosed in adults across nine population-based cancer registries. The study reported a five-year OS rate of 82.8% for female breast cancer.

In the OS, women aged 70 years or older exhibited worse survival compared to the other age groups. This finding is consistent with the literature on survival for female breast cancer^19,20^. The poorer survival may be attributed to advanced age, a higher proportion of late diagnoses, and the fact that standard treatment for breast cancer may not be administered in this age group^19^.

Several studies^19,21,22^ have shown differences in survival between younger women and those over 50 years of age. A study conducted in Iran^20^ found that women over 50 had worse survival outcomes compared to younger women. This study also indicated that the presence of comorbidities leads to secondary disabilities, complicates the treatment of breast cancer, and increases the overall complications associated with cancer in patients.

Education was a variable that showed an association with OS for female breast cancer in the bivariate analysis. Due to the strong correlation between education, unemployment, occupation, and income, this variable can serve as a proxy for a person's socioeconomic status^23,24^.

Several studies^19,21^ from countries with varying human development indices (HDI) have demonstrated better breast cancer survival among women with higher levels of education. In Brazil^23^, the five-year disease-free survival for breast cancer was 85.0% for women with higher levels of education, compared to 70.9% for those with lower levels. In India^24^, the discrepancy was even more pronounced, with OS for women with higher education levels at 94.0%, compared to 61.5% for those with lower education levels.

According to a systematic review conducted by Coughlin^25^, education level and the municipality of residence are associated with breast cancer survival. The review also highlights that poverty, lower levels of education, racial segregation, racial discrimination, lack of racial support, and social isolation are critical factors influencing both the staging of the disease and survival outcomes.

Early diagnosis is a well-established factor related to breast cancer survival^21,22,24,26^, and adherence to screening is one way to achieve this. Several studies have shown an association between marital status^27,28^ and higher education levels with increased adherence to breast cancer screening, including in Brazil^23,29^. In Brazil, sociodemographic variables associated with two years without screening were more prominent among women with lower education levels in the North Region and those living without a partner in the South Region^23^.

Marital status was another variable associated with OS and SS. Some studies have shown that married women have better survival outcomes compared to single/widowed/divorced women^5,27,28,30^. Although the association between marital status and survival varied according to factors such as race/skin color, tumor subtype, and neighborhood socioeconomic status, the strongest association with worse survival was observed among women without a partner living in low-income neighborhoods, even after stratification by tumor subtype^5^.

These findings reflect the association between social inequities and breast cancer, as demonstrated in other studies^25,31^, which show worse survival outcomes for women with lower socioeconomic status.

One limitation of the study is the incompleteness of the RCBP database. According to a previous study^13^, a high number of mandatory variables were missing in the Cancer Hospital Registry (*Registro Hospitalar de Câncer* – RHC) of Mato Grosso, one of the sources that directly feeds the RCBP/Cuiabá. As a result, variables such as staging, which had a high degree of incompleteness in the RHC of Cuiabá, exhibited the same issue in the RCBP database. This made it impossible to analyze the stage of diagnosis, as well as other variables like morphology and extent of the disease, and to examine the association of these variables with education and marital status. However, a major strength of the RCBP database is its availability of historical data on new cases up to 2016, which is more current than other databases in Brazil and Latin America^32^. Another limitation of the study relates to the passive monitoring of new breast cancer cases, which could result in missed deaths that occurred in other states^33^. Additionally, the use of the Kaplan-Meier curve to estimate SS may be less accurate, as it does not account for the conditional independence between the date of diagnosis and time until death for a known set of covariates^34^.

It can be concluded that age group, education level, and marital status influence the OS and SS of female breast cancer, with worse survival observed in women aged 70 years old or older, those with lower education levels, and those without a partner. This finding contributes to the existing evidence from other studies that indicate poorer survival for women with these characteristics.

This research highlights the usefulness of secondary data in profiling individuals treated for breast cancer, as well as in analyzing survival rates for different types of cancer. However, the findings should be considered in the context of the RCBP database's incompleteness, which underscores the need to develop strategies for improving the management of cancer registry data to achieve greater data completeness.

Furthermore, it is necessary to reassess the strategies for controlling female breast cancer in Greater Cuiabá, with the goal of proposing policies focused on early diagnosis, timely treatment, and improving the quality of life for women affected by this pathology.
