# Supplementary material for: Five-year overall and specific survival of breast cancer in great Cuiaba (MT), Brazil
Source: Rev Bras Epidemiol. 2025 Mar 3;28:e250010. doi: 10.1590/1980-549720250010 (PMC11884821; doi:10.1590/1980-549720250010)
Supplement: Translation

# Translation: Sobrevida global e específica de cinco anos do câncer de mama na grande Cuiabá (MT), Brasil

**DOI:** 10.1590/1980-549720250010.2

**Authors:** Jânia Cristiane de Souza Oliveira, Noemi Dreyer Galvão, Amanda Cristina de Souza Andrade, Ageo Mário Cândido da Silva

**Affiliations:** I Universidade Federal de Rondonópolis, Faculdade de Ciências da Saúde – Rondonópolis, (MT), Brasil.; II Universidade Federal de Mato Grosso, Instituto de Saúde Coletiva – Cuiabá (MT), Brasil.; III Secretaria de Estado de Saúde de Mato Grosso – Cuiabá (MT), Brasil.

**Keywords:** Saúde da mulher, Neoplasias da mama, Análise de sobrevida, Sistemas de informação em saúde

## Abstract

**Objetivo:**

Analisar a sobrevida global e específica em cinco anos para o câncer de mama feminino na Grande Cuiabá, Mato Grosso, Brasil.

**Métodos:**

Coorte não concorrente, de base populacional, por meio do Registro de Câncer de Base Populacional da Grande Cuiabá (Cuiabá e Várzea Grande), com mulheres diagnosticadas com câncer de mama no período de 2008 a 2013, seguidas até 2018 no banco de mortalidade regional. A amostra foi composta do total de 1.220 mulheres. Para a análise da sobrevida de cinco anos foram utilizadas as curvas de Kaplan-Meier e o modelo de regressão de riscos proporcionais de Cox, computando-se os *hazard ratios* para a estimativa das variáveis. Compararam-se as curvas por meio do teste *log-rank* (p<0,05). O *linkage* probabilístico foi realizado por meio do *software* Link Plus 2.0, e a análise de sobrevida foi realizada pelo STATA versão 12.0.

**Resultados:**

Não houve diferença estatística entre as sobrevidas global (SG) e a específica (SE) (SG 78,0%, intervalo de confiança de 95% — IC95% 75,6–80,2; SE 81,0%, IC95% 78,7–83,2). Apresentaram pior sobrevida as mulheres com menor escolaridade (SG 58,33%; SE 64,89%) e sem parceiro (SG 64,81%; SE 70,41%).

**Conclusão:**

Observa-se, assim, que a escolaridade e o estado civil afetaram as sobrevidas global e específica para o câncer de mama feminino. Faz-se necessário propor políticas que atendam ao perfil de mulheres com menor sobrevida.

## INTRODUÇÃO

O câncer de mama é o mais incidente na população feminina, no mundo. Em 2022, a taxa estimada de casos novos de câncer de mama foi de 46,8/100.000 mulheres. Para 2045, estima-se aumento de 46,5%, com mais de 3,3 milhões de mulheres diagnosticadas com essa patologia. Para a América do Sul, é estimado um aumento de 47,8% casos novos para o mesmo período^1^.

Mundialmente, esta também é a primeira causa de óbito por câncer em mulheres, com taxa de 12,7/100.000 mulheres em 2022, com aumento estimado de 59,1% nos óbitos no mundo até 2045. Para os países da América do Sul, a taxa estimada para 2022 foi de 13,8/100.000 mulheres e projeta-se aumento de 63,3% nos óbitos até 2045^1,2^.

No Brasil, trata-se do câncer mais incidente em mulheres em todas as regiões do país. Para o triênio 2023–2025, estimou-se taxa de incidência ajustada de 66,5/100.000 mulheres e aumento de 47,6% até 2045. Para o ano de 2022, estimou-se taxa de mortalidade ajustada de 13,9/100.000 mulheres e aumento estimado de 61,9% até 2045 na mortalidade por câncer de mama no país^2,3^.

A sobrevida para o câncer nas Américas é heterogênea. Na América do Norte e em Costa Rica, a sobrevida é superior à dos demais países (≥85,0%). Na própria América Latina, observam-se disparidades. Enquanto Argentina, Peru e Porto Rico apresentaram sobrevida entre 80,0 e 84,0%, países como Brasil, Colômbia, Cuba e Equador estão entre aqueles com sobrevida de 70,0 a 79,0%^4^.

Além do diagnóstico precoce, múltiplos fatores contribuem para a melhor sobrevida de mulheres com câncer de mama, tais como *status* receptor hormonal positivo, diagnóstico de câncer de mama pós-menopausa, não possuir subtipo triplo negativo, raça/cor da pele branca, mulheres com parceiro, com *status* socioeconômico mais elevado, possuir seguro (plano de saúde)^5-7^.

Outro fator é que, apesar de o câncer de mama feminino compor uma das prioridades do Plano Oncológico estadual, seu controle ainda é um desafio tanto em termos de melhoria da articulação da rede quanto de investimento para diminuir o intervalo entre as primeiras suspeitas, o diagnóstico da doença e o início do tratamento. Ademais, o estudo fornecerá informações quanto à realidade para subsidiar futuras ações para o controle da doença^8^.

Dessa maneira, o objetivo do estudo foi analisar a sobrevida global (SG) e específica (SE) em cinco anos para o câncer de mama feminino, na coorte de 2008 a 2013, conforme a base populacional da Grande Cuiabá, Mato Grosso, Brasil.

## MÉTODOS

Foi constituída coorte não concorrente, de base populacional, com mulheres diagnosticadas com câncer de mama, inseridas no Registro de Câncer de Base Populacional — RCBP/Cuiabá, composto dos municípios de Cuiabá e Várzea Grande, estado de Mato Grosso, Brasil, no período de janeiro de 2008 a dezembro de 2013. Foram selecionados apenas os casos incidentes (indicador de definitivo "*true*") e excluídos os casos de câncer de mama *in situ*. O seguimento foi realizado de maneira passiva no Sistema de Informação sobre Mortalidade (SIM) Mato Grosso, até completar cinco anos.

A cobertura do RCBP Cuiabá inclui os municípios de Cuiabá e Várzea Grande, os dois maiores municípios do estado, contíguos, com população de quase um milhão de habitantes segundo o censo de 2022, com 650.877 habitantes em Cuiabá^9^ e 300.078 em Várzea Grande; o conglomerado urbano é denominado de Grande Cuiabá^10^.

O RCBP de Cuiabá foi fundado em 1999 pela Secretaria de Estado de Saúde de Mato Grosso. Os dados estavam desatualizados desde 2007. Por isso, em 2016, iniciou-se uma parceria com a Universidade Federal de Mato Grosso para a atualização dos bancos, que perdurou até março de 2021. Atualmente, o RCBP Cuiabá conta com 38 estabelecimentos de saúde como fontes notificadoras, entre hospitais e clínicas de apoio e diagnóstico^11^. Deles, três hospitais estão no cadastro do INCA como Unidade de Alta Complexidade em Oncologia (Unacon)^12^. Quanto aos Registros Hospitalares de Câncer, que alimentam o RCBP Cuiabá, eles ainda apresentam uma incompletude elevada de variáveis relevantes, tais como escolaridade, estado civil, tumor-nódulo-metástase (TNM), estadiamento e estado da doença ao fim do primeiro tratamento^13^.

Quanto ao preparo dos bancos, anteriormente ao pareamento entre os bancos, foi realizada uma limpeza no banco do SIM, excluindo-se os casos cujo nome do óbito estivesse preenchido como "em branco", "desconhecido", "ignorado" ou "indigente", com o total de 72 exclusões. No banco do RCBP, foram removidas as duplicidades. Foram classificados como duplicidades os registros que apresentassem dados idênticos de nome do paciente, nome da mãe, sexo, data de nascimento, código da doença e data de diagnóstico, totalizando 18 exclusões (Figura 1).

**Figura 1:** Fluxograma.

O *linkage* probabilístico entre os bancos RCBP e SIM foi realizado seguindo-se os três passos propostos por Coeli e Camargo Jr.^14^, quais sejam: padronização, uniformização dos campos comuns a serem empregados no pareamento; blocagem, por meio da variável sexo; e, finalmente, o pareamento por meio da construção de escores de concordância baseados nas variáveis nome do paciente (óbito), nome da mãe e data de nascimento.

Com base no relacionamento probabilístico entre os bancos, foram encontrados 273 pares, todos verdadeiros. Destes, cinco registros não possuíam informação de "idade" e "data de nascimento", e um apresentou idade igual a zero (data de nascimento igual à data de diagnóstico). Após a tentativa de recuperar esses dados no Sistema de Cadastramento de Usuários do Sistema Único de Saúde (CADSUS), foi possível recuperar apenas o registro da idade igual a zero. Os demais sem informação de idade foram excluídos do banco, totalizando 1.220 registros: sendo 268 falhas (mulheres que apresentaram o desfecho óbito durante o período do estudo) e 952 censuras (mulheres que não apresentaram o desfecho óbito durante o período do estudo)^15^ (Figura 1).

A variável dependente foi o tempo de entrada das mulheres no estudo (data do diagnóstico por câncer de mama) até o óbito por qualquer causa (SG) ou por câncer de mama (SE). Estudos assim são denominados análise de sobrevida^15^. O tempo de sobrevida foi computado em meses, e as mulheres que não apresentaram o evento de interesse durante o período do estudo foram consideradas censuras.

Foram analisadas as seguintes variáveis independentes: faixa etária (20–49 anos, 50–69 anos, ≥70 anos); raça/cor da pele (amarela, branca, parda, preta e sem informação); estado civil (com parceiro, sem parceiro e sem informação); escolaridade 0–7, 8+ anos de estudo e sem informação); município de residência no momento do diagnóstico da doença (Cuiabá, Várzea Grande); meio diagnóstico (histologia do tumor primário, outros) e morfologia (carcinoma ductal infiltrante, outros).

Realizou-se uma avaliação inicial por meio de frequências absolutas e relativas. As variáveis escolaridade, estado civil e raça/cor da pele apresentaram 42,70, 26,48 e 9,75% de dados sem informação, respectivamente. Diante da impossibilidade de imputação múltipla dos dados faltantes pelo número reduzido de variáveis independentes disponíveis e com dados completos^16^, realizou-se o modelo múltiplo somente com dados completos (excluindo os sem informação) (n=645).

Foram calculadas as probabilidades de sobrevida e seus respectivos intervalos de 95% de confiança (IC95%) conforme as variáveis independentes. Para estimar as curvas de sobrevida foi utilizado o estimador de Kaplan-Meier para o evento de interesse, e as curvas foram comparadas entre si por meio do teste de *log-rank*, com nível de significância de 5%.

Para estimar o efeito das variáveis independentes na sobrevida, utilizou-se o modelo de regressão de riscos proporcionais de Cox, computando-se os *hazard ratios* (HR) e correspondentes IC95%. As variáveis morfologia e meio diagnóstico não atenderam ao pressuposto de proporcionalidade de riscos de Cox, por isso o modelo múltiplo foi estratificado por essas variáveis. A pressuposição de proporcionalidade dos riscos foi verificada por meio dos resíduos de Schoenfeld, desde que não houvesse rejeição da hipótese nula (p>0,05)^16^.

Utilizou-se o *software* Link Plus 2.0 para a realização do *linkage* probabilístico e Stata 12.0 para a análise de sobrevida.

O estudo atende aos preceitos da Resolução n° 466/2012 do Conselho Nacional de Saúde, pois compõe o projeto matricial intitulado "Câncer e seus fatores associados: análise de registro base populacional e hospitalar de Cuiabá-MT", aprovado pelo Comitê de Ética do Hospital Universitário Júlio Muller e do Comitê de Ética da Escola de Saúde Pública do Estado de Mato Grosso — Secretaria de Estado de Saúde de Mato Grosso.

## RESULTADOS

Das 1.220 mulheres do estudo, 80,08% residiam em Cuiabá, 59,67% eram de raça/cor não branca, 29,75% possuíam mais de oito anos de estudo, 36,80% estavam sem parceiro, 92,62% foram diagnosticadas por meio da histologia do tumor primário e 78,85% foram diagnosticadas com a morfologia carcinoma ductal invasivo. A maioria das mulheres tinha 50 anos ou mais (60,33%), a média de idade foi de 54 anos, a mediana foi de 53 anos (intervalo interquartílico de 45 a 63 anos), variando de 21 a 99 anos de idade (Tabela 1).

**Tabela 1:** Caracterização das mulheres diagnosticadas com câncer de mama. Cuiabá (MT), 2008–2013.

Variável		Total n=1.220 (%)	Óbito por qualquer causa n=268 (%)	Óbito por câncer de mama n=228 (%)	Censura n=952 (%)
Faixa etária (anos)					
	20–49	484 (39,67)	99 (36,94)	90 (39,47)	385 (40,45)
	50–69	572 (46,89)	114 (42,54)	99 (43,42)	458 (48,11)
	+ 70	164 (13,44)	55 (20,52)	39(17,11)	109 (11,44)
Raça/cor					
	Amarela	36 (2,95)	4 (1,49)	3 (1,32)	32 (3,36)
	Branca	373 (30,57)	94 (35,07)	75 (32,89)	279 (33,41)
	Parda	635 (52,05)	150 (55,97)	132 (57,89)	485 (50,95)
	Preta	57 (4,67)	18 (6,72)	16 (7,02)	39 (4,10)
	Sem informação	119 (9,75)	2 (0,75)	2 (0,87)	117 (12,28)
Escolaridade (anos)					
	0–7	336 (27,54)	140 (52,24)	114 (50,00)	196 (20,59)
	8+	363 (29,75)	116 (43,28)	103 (45,18)	247 (25,95)
	Sem informação	521 (42,70)	12 (4,48)	11 (4,82)	509 (53,47)
Estado civil					
	Com parceiro	448 (36,72)	106 (39,55)	95 (41,67)	342 (35,92)
	Sem parceiro	449 (36,80)	158 (58,96)	129 (56,58)	291 (30,57)
	Sem informação	323 (26,48)	4 (1,49)	4 (1,75)	319 (33,51)
Município					
	Cuiabá	977 (80,08)	216 (80,60)	181 (79,39)	761 (79,94)
	Várzea Grande	243 (19,92)	52 (19,40)	47 (20,61)	191 (20,06)
Meio diagnóstico					
	Histologia do tumor primário	1.130 (92,62)	219 (81,72)	179 (78,51)	911 (95,69)
	Outros	88 (7,21)	49 (18,28)	49 (21,49)	39 (4,10)
	Sem informação	2 (0,16)	0	0	2 (0,21)
Morfologia					
	Carcinoma ductal invasivo	962 (78,85)	184 (68,66)	155 (67,98)	778 (81,72)
	Outros	258 (21,15)	84 (31,34)	73 (32,02)	174 (18,28)

Fonte: Dados da pesquisa; 2021.

Até o término do estudo, das 1.220 mulheres em seguimento, 268 foram a óbito, sendo 228 (85,76%) por câncer de mama, 14 (5,23%) por outro tipo de câncer e 26 (9,71%) por outras causas identificadas.

A SG e a SE não apresentaram diferença estatística significante (SG 78,03, IC95% 75,60–80,25%; SE 81,05, IC95% 78,72–83,16%). Na SG, a faixa etária igual ou maior que 70 anos e a escolaridade de zero a sete anos apresentaram sobrevida menor e as mulheres sem parceiro apresentaram menor SG e SE (teste *log-rank* p<0,05) (Tabela 2 e Figura 2). A histologia do tumor primário apresentou as maiores SG e SE entre os meios diagnósticos e o carcinoma ductal invasivo quanto à morfologia (p<0,05) (Tabela 2).

**Tabela 2:** Funções de sobrevida global e sobrevida específica de cinco anos das mulheres diagnosticadas com câncer de mama, segundo as variáveis do estudo. Cuiabá (MT), 2008–2013.

Variável		Sobrevida global % (IC95%)	Sobrevida específica % (IC95%)
Faixa etária (anos)			
	20–49	79,55 (75,67–82,87)	81,17 (77,37–84,40)
	50–69	80,07 (76,56–83,12)	82,51 (79,12–85,40)
	+ 70	66,46 (58,68–73,12)	75,58 (68,12–81,53)
Raça/cor			
	Amarela	88,89 (73,05–95,68)	91,43 (75,73–97,15)
	Branca	74,80 (70,07–78,90)	79,47 (74,95–83,27)
	Pardo	76,38 (72,88–79,49)	78,96 (75,56–81,95)
	Preto	68,42 (54,66–78,79)	71,55 (57,82–81,50)
	Sem informação	98,32 (93,45–99,58)	98,32 (93,45–99,58)
Escolaridade (anos)			
	0–7	58,33 (52,87–63,39)	62,87 (54,92–69,82)
	8+	68,04 (62,98–72,57)	73,83 (66,29–79,94)
	Sem informação	97,66 (96,01–98,64)	97,84 (96,23–98,77)
Estado civil			
	Com parceiro	85,73 (83,06–88,01)	87,09 (84,51–89,27)
	Sem parceiro	64,81 (60,20–69,03)	70,41 (65,87–74,47)
	Sem informação	98,76 (96,73–99,53)	98,76 (96,73–99,53)
Município			
	Cuiabá	77,89 (75,16–80,37)	81,21 (78,60–83,54)
	Várzea Grande	78,60 (72,89–83,25)	80,41 (74,80–84,90)
Meio diagnóstico			
	Histologia do tumor primário	80,62 (78,19–82,81)	83,87 (81,57–85,91)
	Outros	44,32 (33,79–54,32)	44,32 (33,79–54,32)
Morfologia			
	Carcinoma ductal invasivo	80,97 (78,24–82,22)	83,62 (81,10–85,89)
	Outros	67,44 (61,35–72,79)	71,52 (65,56–76,63)

Fonte: Dados da pesquisa, 2021.

**Figura 2:** Sobrevida global e sobrevida específica de cinco anos do câncer de mama feminino, Registro de Câncer de Base Populacional — RCBP/Cuiabá (MT), Brasil.

RCBP: Registro de Câncer de Base Populacional; SIM: Sistema de Informações sobre Mortalidade.

Para o modelo ajustado de Cox, a faixa etária apresentou significância estatística somente para a SG. Para os modelos ajustados, incluindo o Cox estratificado, o estado civil apresentou significância estatística para ambas as sobrevidas. As demais variáveis não apresentaram diferença estatisticamente significativa (Tabela 3).

**Tabela 3:** *Hazard ratios* (HR) não ajustados e ajustados para sobrevida global e sobrevida específica de cinco anos das mulheres diagnosticadas com câncer de mama, segundo as variáveis selecionadas para o modelo múltiplo. Cuiabá (MT), 2008–2013.

Variáveis			Não ajustada	Análise ajustada	
				Modelo COX*,‡	Modelo COX estratificado†,‡
			HR (IC95%)	HR (IC95%)	HR (IC95%)
Sobrevida global					
	Faixa etária (anos)				
		20–49	1	1	1
		50–69	0,98 (0,75–1,28)	0,97 (0,73–1,28)	0,94 (0,71–1,25)
		+ 70	1,78 (1,28–2,47)	1,55 (1,08–2,25)	1,29 (0,89–1,87)
	Raça/cor§				
		Amarela	1	1	1
		Branca	0,95 (0,35–2,58)	0,91 (0,33–2,49)	0,76 (0,28–2,09)
		Parda	0,81 (0,30–2,20)	0,79 (0,29–2,16)	0,67 (0,24–1,82)
		Preta	1,20 (0,41–3,55)	1,09 (0,37–3,25)	0,98 (0,33–2,91)
	Escolaridade// (anos)				
		0–7	1,40 (1,09–1,79)	1,24 (0,95–1,60)	1,23 (0,94–1,60)
		8+	1	1	1
	Estado civil¶				
		Com parceiro	1	1	1
		Sem parceiro	1,59 (1,24–2,03)	1,44 (1,11–1,87)	1,49 (1,15–1,94)
	Município				
		Cuiabá	1,03 (0,76–1,40)	1,02 (0,75–1,41)	1,01 (0,73–1,38)
		Várzea Grande	1	1	1
Sobrevida específica					
	Faixa etária (anos)				
		20–49	1	1	1
		50–69	0,93 (0,70–1,24)	0,93 (0,69–1,25)	0,89 (0,66–1,21)
		+70	1,38 (0,95–2,01)	1,22 (0,80 –1,87)	0,99 (0,64–1,51)
	Raça/cor§				
		Branca	1	1	1
		Amarela	0,99 (0,31–3,17)	0,97 (0,30–3,11)	0,80 (0,25–2,58)
		Parda	0,95 (0,30–2,98)	0,92 (0,29–2,91)	0,76 (0,24–2,41)
		Preta	1,42 (0,41–4,87)	1,34 (0,39–4,62)	1,23 (0,35–4,24)
	Escolaridade// (anos)				
		0–7	1,32 (0,88–1,97)	1,17 (0,88–1,55)	1,16 (0,87–1,53)
		8+	1	1	1
	Estado civil¶				
		Com parceiro	1	1	1
		Sem parceiro	1,45 (1,11–1,89)	1,37 (1,03–1,81)	1,44 (1,09–1,91)
	Município				
		Cuiabá	0,96 (0,69–1,32)	0,97 (0,69–1,36)	0,95 (0,68–1,35)
		Várzea Grande	1	1	1

Fonte: Dados da pesquisa, 2021.

* Valor global do teste de suposição de riscos proporcionais p=0,733 (SG); p=0,285 (SE);

† Valor global do teste de suposição de riscos proporcionais p=0,853 (SG); p=0,754 (SE); Estratificado pelas variáveis morfologia e meio diagnóstico;

‡ n=645;

§ n=1,010;

// n=699;

¶ n=897.

## DISCUSSÃO

Neste estudo, analisou-se a SG e a SE para o câncer de mama feminino na Grande Cuiabá, Mato Grosso, Brasil. Não houve diferença estatística entre as duas. Na análise múltipla a variável estado civil apresentou significância estatística para ambas as sobrevidas (p<0,05).

O achado do nosso estudo foi semelhante à sobrevida encontrada para o Brasil (dados dos RCBP de Aracaju, Cuiabá, Curitiba, Goiânia, Jaú e São Paulo), realizado pelo CONCORD-3^4^, que comparou a sobrevida líquida de 18 cânceres em 71 países. Em países como Dinamarca, França, Noruega, Finlândia, Israel, Japão, Austrália, Nova Zelândia, Canadá, Estados Unidos e Costa Rica, a sobrevida para o câncer de mama feminino foi mais elevada (≥85,0%). Argentina, Peru e Porto Rico apresentaram sobrevida de 80,0–84,0%. O Brasil está entre os países com sobrevida de 70,0–79,0%, como Cuba, Equador, Bulgária e Polônia. Em outro estudo realizado na Polônia, no período de 2000 a 2019, a sobrevida foi de 77,3% em cinco anos^17^, resultado semelhante ao observado neste estudo.

Estudo de base populacional realizado em Goiânia^18^, com mulheres diagnosticadas com câncer de mama entre 1995 e 2003, mostrou SG de 72,1% em cinco anos, menor que a encontrada no presente estudo. Esta diferença talvez seja devida ao período do estudo, ou ainda às mudanças no rastreamento e protocolo de tratamento da doença.

Melhor sobrevida foi observada na Espanha^19^, coorte de 2000 a 2007, que analisou todos os tumores primários possíveis de diagnóstico em adultos, em nove registros de câncer de base populacional, com SG de 82,8% em cinco anos para o câncer de mama feminino.

Na SG, a faixa etária de 70 anos ou mais apresentou pior sobrevida se comparada às demais. O achado quanto à idade é consistente com a literatura acerca da sobrevida para o câncer de mama feminino^19,20^. Talvez isso seja decorrente da idade avançada e da maior proporção de diagnóstico tardio, além do fato de não ser feito o tratamento padrão para o câncer de mama^19^.

Vários estudos^19,21,22^ revelaram diferença na sobrevida entre mulheres jovens e acima dos 50 anos de idade. Em pesquisa realizada no Irã^20^, a sobrevida das mulheres com mais de 50 anos foi pior que a das mais jovens. O estudo demonstrou ainda que a presença de comorbidades causa deficiências secundárias e afeta o tratamento da doença, além de potencializar as complicações do câncer em pacientes.

A escolaridade foi uma variável que, na análise bivariada, apresentou associação com a SG para o câncer de mama feminino. Dada a forte correlação entre escolaridade, desemprego, ocupação e renda, essa variável pode ser utilizada como *proxy* do *status* socioeconômico de uma pessoa^23,24^.

Alguns estudos^19,21^ de países com baixo, médio e alto índice de desenvolvimento humano (IDH) mostraram melhor sobrevida de câncer de mama para as mulheres com maior escolaridade. No Brasil^23^, a sobrevida livre de doença para o câncer de mama em cinco anos em mulheres com maior escolaridade foi de 85,0% e, para as mulheres com menor escolaridade, de 70,9%. Na Índia^24^, houve discrepância ainda mais acentuada, com SG para as mulheres com maior escolaridade de 94,0% e para as de menor escolaridade de 61,5%.

De acordo com a revisão sistemática conduzida por Coughlin^25^, escolaridade e o distrito municipal de residência estão associados à sobrevida por câncer de mama. Igualmente, o estudo revela que pobreza, menos anos de estudo, segregação racial, discriminação racial, falta de suporte racial e isolamento social são fatores críticos para o estadiamento da doença, assim como para a sobrevida dessa patologia.

O diagnóstico precoce é um fator já bem conhecido relacionado à sobrevida de câncer de mama^21,22,24,26^. A adesão ao rastreamento é uma maneira de alcançá-lo. Alguns estudos têm demonstrado associação entre o estado civil casada^27,28^ e maior escolaridade, com maior adesão ao rastreamento ao câncer de mama, até mesmo no Brasil^23,29^. Para as mulheres brasileiras, as variáveis sociodemográficas associadas com dois anos sem rastreamento foram mais salientes em mulheres com menor escolaridade na Região Norte e naquelas que vivem sem parceiro na Região Sul^23^.

Outra variável que apresentou associação com as SG e SE foi o *status* marital. Em alguns estudos, foi demonstrado que mulheres casadas apresentaram melhor sobrevida que as solteiras/viúvas/divorciadas^5,27,28,30^. Apesar da associação do *status* marital variar de acordo com a raça/cor da pele, o subtipo de tumor e o *status* socioeconômico da vizinhança, a associação mais elevada para pior sobrevida foi observada entre as mulheres sem parceiro que residem em vizinhança de baixa renda, mesmo após a estratificação por subtipo do tumor^5^.

Estes achados refletem a associação entre as iniquidades sociais e o câncer de mama, como demonstrado em outros estudos^25,31^, com pior sobrevida para mulheres com pior nível socioeconômico.

Uma limitação da pesquisa é a incompletude do banco do RCBP. De acordo com estudo prévio^13^, um número elevado de variáveis obrigatórias foi observado no Registro Hospitalar de Câncer (RHC) de Mato Grosso, uma das fontes que alimenta diretamente o RCBP/Cuiabá. Deste modo, variáveis como estadiamento, por exemplo, que apresentaram elevado grau de incompletude no RHC de Cuiabá, apresentaram a mesma característica no banco RCBP, o que impossibilitou a análise do estágio de diagnóstico da doença, além de análises de outras variáveis como morfologia e extensão da doença. Impediu ainda a análise de associação dessas variáveis com a escolaridade e o *status* marital. Entretanto, um grande benefício do banco RCBP é sua disponibilidade de série histórica de casos novos até 2016, mais atual que outros bancos do Brasil e da América Latina^32^. Outra limitação do estudo se refere ao seguimento passivo dos casos novos de câncer de mama, que pode levar à não identificação de óbitos ocorridos em outros estados^33^. Também foi uma limitação do estudo estimar a curva de Kaplan-Meier por meio da SE, o que pode não ser tão preciso, uma vez que não considera que a data de diagnóstico e o tempo até o óbito são condicionalmente independentes para um conjunto conhecido de covariáveis^34^.

Pode-se concluir que a faixa etária, a escolaridade e o estado civil afetam a SG e SE do câncer de mama feminino, com pior sobrevida para as mulheres com 70 anos ou mais, com menor escolaridade e sem parceiro. Este achado soma-se às evidencias de outros estudos, que apontam pior sobrevida para mulheres com essas características.

Esta pesquisa realça a utilidade de dados secundários para traçar o perfil de pessoas atendidas além da análise de sobrevida de cada tipo de câncer. Todavia, os achados precisam considerar o uso de um banco do RCBP, com incompletude, que apontam para a necessidade de desenvolver estratégias de melhoria da gestão da informação dos registros de câncer para maior completitude dos dados.

Ademais, faz-se necessário rever as estratégias de controle do câncer de mama feminino na Grande Cuiabá, com vistas a propor políticas visando ao diagnóstico precoce, ao tratamento oportuno e à melhoria da qualidade de vida das mulheres perante esta patologia.
